# Pseudochylothorax Combined with Spontaneous Pneumothorax: Case Report of a Rare Complication of Rheumatoid Arthritis

**DOI:** 10.1155/2018/7846962

**Published:** 2018-04-22

**Authors:** Raquel Rosa, Dionísio Maia, Nídia Caires, Rita Gerardo, Inês Gonçalves, João Cardoso

**Affiliations:** Pulmonology Department, Hospital de Santa Marta, Centro Hospitalar de Lisboa Central, Lisbon, Portugal

## Abstract

Pleural involvement is the most frequent thoracic complication of rheumatoid arthritis (RA), usually occurring in patients with known RA. Typical rheumatoid pleural effusion is an exudate characterized by low pH and glucose levels and high LDH activity. Rarely, it has features of pseudochylothorax. Other uncommon complications are pneumothorax, hydropneumothorax, empyema, and bronchopleural fistula. The case of a 51-year-old man with a spontaneous, small, and asymptomatic hydropneumothorax with features of pseudochylothorax is presented. After careful clinical and laboratory evaluation, he was diagnosed with rheumatoid arthritis, and we admitted that the pleural changes were secondary to the connective tissue disease. He started immunosuppressive treatment and maintained stability during follow-up, without need of specific pleural treatment. We hypothesized that the pleural nodule found on the chest computed tomography scan was related with the simultaneous occurrence of pleural effusion and pneumothorax. This is a rare presentation and complication of RA, highlighting the utility of a comprehensive clinical and laboratory evaluation and focusing on the importance of pleural rheumatoid nodules in the pathogenesis of RA pleural disease.

## 1. Introduction

Pleural disease is the most frequent intrathoracic manifestation of rheumatoid arthritis (RA) and causes pleural effusion in up to 20% of patients, although clinically apparent in only 5% [1]. Pleural rheumatoid effusion is typically small, unilateral, asymptomatic, and more commonly found in a middle-aged man with long-standing active arthritis, subcutaneous rheumatoid nodules, and high titers of rheumatoid factor. The effusion is an exudate with high LDH (>700 U/L), low pH (<7.2), and very low glucose (<50 mg/dL in 75% of cases). Elevated values of adenosine deaminase can be found. In rare cases, cholesterol concentration is increased (pseudochylothorax). A pleural fluid differential cell count usually reveals predominance of neutrophils in the acute phase followed by lymphocytes in chronicity. Its cytology may demonstrate the triad of giant multinucleated histiocytes, elongated macrophages, and necrotic background material. Pleural biopsy is usually not needed for diagnosis, but the presence of classic rheumatoid nodules on the pleura is pathognomonic for RA [1, 2]. Most cases of pleural involvement do not require specific treatment as they are asymptomatic and/or resolve spontaneously or with underlying RA treatment. Pneumothorax, hydropneumothorax, empyema, and bronchopleural fistula are other uncommon RA pleural complications, and the treatment can be challenging [3]. We report the case of a middle-aged man presenting with pseudochylothorax and spontaneous pneumothorax leading to the diagnosis of RA.

## 2. Case Report

**Figure 1:**
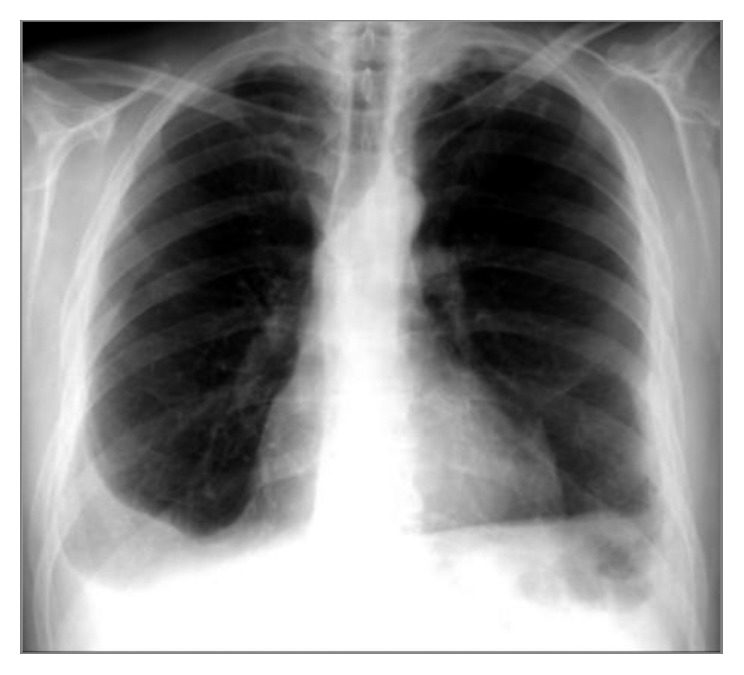
Chest radiography showing right-sided pleural effusion and left diaphragmatic retraction.

A 51-year-old man was admitted to the hospital for investigation of an asymptomatic right pleural effusion found on routine chest radiography (Figure 1). He was a current smoker (44 pack years) and had had tuberculosis at the age of 36, treated with first line drugs during one year. There was no other significant past medical history, and he was not taking any medication. His family history was irrelevant.

On physical examination, he was found to have clinical features of a small right-sided pleural effusion. Blood tests revealed mild chronic/inflammatory anemia (Hb 12.4 g/dL) and leukocytosis (13.3 × 10^9^/L) without neutrophilia; the erythrocyte sedimentation rate (34 mm/h) and the C-reactive protein level (27.6 mg/L) were elevated. Chest radiography also showed left diaphragm retraction (Figure 1).

**Figure 2:**
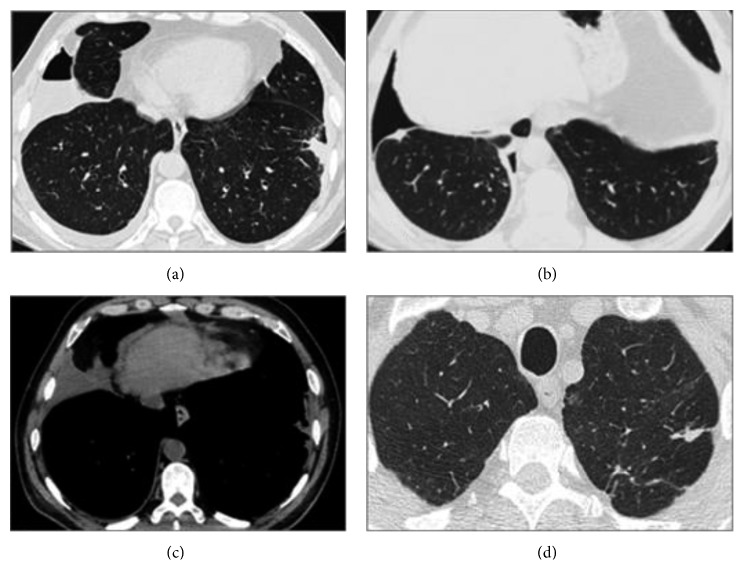
Computed tomography scan of the chest showing on the right lung: (a) hydropneumothorax, (b) basal pleural nodule, and (c) pleural thickening; on the left lung: (a) basal pleural residual changes and (d) upper lobe residual parenchymal changes consistent with past tuberculosis.

A computed tomography scan of the chest confirmed the presence of a right pleural effusion with associated pleural thickening and showed ipsilateral small basal pneumothorax; a pleural nodule was noted. On the left, a slight focal basal pleural thickening and upper lobe residual parenchymal changes were consistent with past tuberculosis (Figure 2). There was no evidence of tuberculosis reactivation, lung consolidation, nodules or masses, emphysema, or enlarged mediastinal lymph nodes.

Through thoracentesis, a milky fluid was obtained, with very low pH (7.0). Empyema was suspected, and the patient was treated accordingly, with chest tube drainage and intravenous broad-spectrum antibiotics. Biochemical fluid analysis revealed an exudate (proteins 68.9 g/L; lactate dehydrogenase (LDH) 2986 U/L) with very low glucose (<10 mg/dL), high adenosine deaminase (201 U/L), and high cholesterol (205 mg/dL). On microscopic examination, cholesterol crystals were observed. Gram and Ziehl–Neelsen stains were negative. Pleural fluid cytology was normal, and blind pleural biopsies revealed fibrosis and marked inflammatory infiltrate without granulomas or neoplastic tissue. The diagnosis of pseudochylothorax was then established.

**Figure 3:**
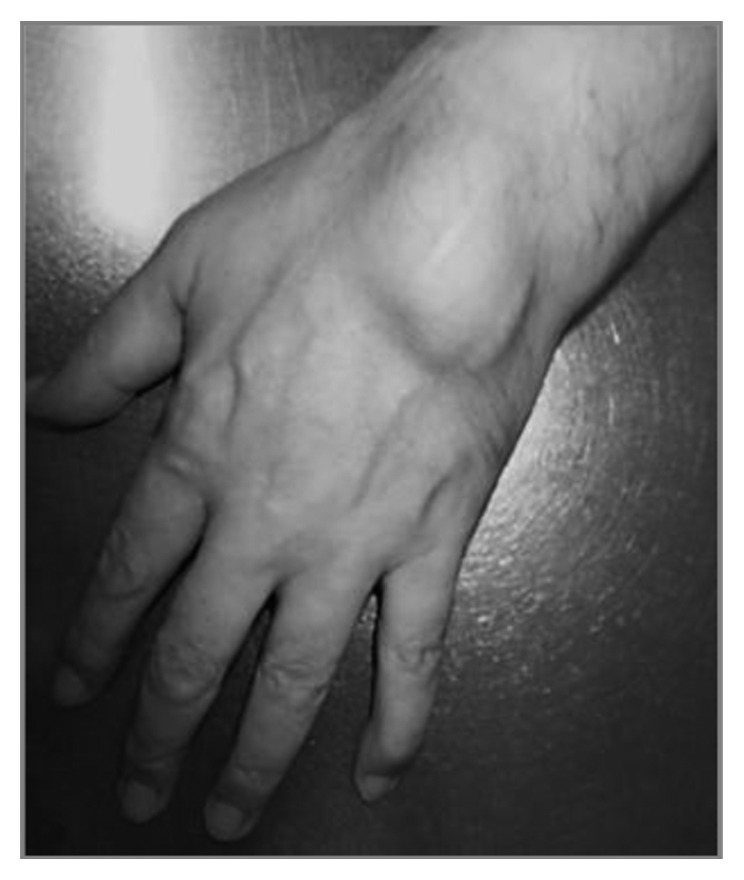
Subcutaneous rheumatoid arthritis nodule.

After careful anamnesis, the patient revealed complains of pain and swelling of multiple joints (hands, wrists, elbows, and knees) for some years. Subcutaneous nodules were seen on his left elbow and wrist (Figure 3), which proved to be RA nodules after surgical excision.

Rheumatoid factor was weakly positive (31.3 UI/mL), and anti-cyclic citrullinated peptide antibody was highly positive (>250 UA/mL). According to 2010 American College of Rheumatology/European League Against Rheumatism (ACR-EULAR) classification criteria [4], the patient was diagnosed with RA.

The antibiotics were stopped after seven days, the chest tube was removed, and the patient was discharged home. Immunosuppressive treatment was initiated at rheumatology outpatient clinic, with improvement of the articular symptoms. Two years after the diagnosis, the patient has no respiratory symptoms, pneumothorax has not recurred, and pleural effusion is stable without any additional therapy directed at the pleural space.

## 3. Discussion

To the best of our knowledge, this is the second reported case of the simultaneous occurrence of pseudochylothorax and pneumothorax in a patient presenting with rheumatoid arthritis [5]. Other cases of hydropneumothorax have been documented in RA patients, but none with accompanying pseudochylothorax [6–11].

In our case, pleural fluid biochemical features and the patient's past of tuberculosis and smoking habits raised suspicion for tuberculosis relapse or cancer. On the other hand, we decided to maintain treatment for empyema until pleural infection was ruled out. The negative pleural fluid cultures and unspecific pleural biopsies in the absence of classic clinical and radiological picture of tuberculosis, cancer or empyema, were helpful. Rheumatoid nodules were not found on blind pleural biopsies, but thoracoscopy was not performed as the diagnosis of RA was established and the pleural effusion resolved. The diagnosis was based on the finding of pseudochylothorax in a patient with articular complaints, positive rheumatoid factor, positive anti-cyclic citrullinated peptide antibody, and pathology-proven subcutaneous RA nodules. The chest computed tomography scan showed a pleural nodule we speculate to be a rheumatoid nodule, probably explaining the simultaneous occurrence of pseudochylothorax (in the absence of significant pleural thickening) and pneumothorax.

Pseudochylothorax (chyliform or cholesterol pleurisy) is a rare pleural effusion with milky appearance because of its high lipid content. Typically, the pleural fluid cholesterol level is greater than 200 mg/dL, the triglyceride level is below 110 mg/dL, the cholesterol/triglycerides ratio is >1, and cholesterol crystals are present [12, 13]. The last two parameters seem to be the most sensitive to establish the diagnosis [13]. Tuberculosis is by far its most frequent cause, followed by RA. A recent systematic review revealed that they account together for 88.5% of all cases of pseudochylothorax (tuberculosis 50.6% and RA 37.9%). Less than 40 cases of RA-associated pseudochylothorax have been reported in the literature [13].

The reason why pseudochylothorax occurs in RA remains unknown. It was thought that it could only develop in patients with chronic pleural effusions (usually of five years' duration or longer) and thickened or calcified pleura [14]. In these circumstances, cholesterol is filtrated into the pleural space during inflammation, and it also originates from the lysis of erythrocytes and neutrophils, being trapped within the pleural space as it cannot be reabsorbed. However, in recent years, some cases have been reported with minimal or without pleural thickening, putting it out as a key factor of pseudochylothorax etiopathogenesis [15–17].

Some authors suggested the hypothesis that rheumatoid pleural effusions develop as an inflammatory response to the presence of rheumatoid nodules [10, 11]. Those nodules are infrequently seen in RA population (1%) and, like pleural effusions, are usually associated with long-standing seropositive RA, male sex, and subcutaneous rheumatoid nodules. The nodules are subpleural or pleural in distribution, vary in size, and are mostly asymptomatic. The cavitation and rupture of these lesions into the pleura space may occur due to their large amounts of proteolytic enzymes. The result can be a pneumothorax, hydropneumothorax, empyema, or bronchopleural fistula [2].

This case illustrates a very unusual presentation and complication of rheumatoid pleural disease. It suggests that when a pleural effusion is detected and has pseudochylothorax features (even in the absence of marked pleural thickening), the diagnosis of RA should be considered, particularly in patients with articular complaints. The simultaneous occurrence of pneumothorax highlights the importance of rheumatoid nodules in the pathogenesis of RA pleural disease.

## References

[1] Balbir-Gurman A., Yigla M., Nahir A. M., Braun-Moscovici Y. (2006). Rheumatoid pleural effusion. *Seminars in Arthritis and Rheumatism*.

[2] Cosgrove G., Schwarz M., Grippi M. A., Elias J. A., Fishman J. A., Kotloff R. M., Pack A. I., Senior R. M. (2015). Pulmonary manifestations of the collagen vascular diseases. *Fishman’s Pulmonary Diseases and Disorders*.

[3] Chansakul T., Dellaripa P. F., Doyle T. J., Madan R. (2015). Intra-thoracic rheumatoid arthritis: imaging spectrum of typical findings and treatment related complications. *European Journal of Radiology*.

[4] Aletaha D., Neogi T., Silman A. J. (2010). 2010 rheumatoid arthritis classification criteria: an American College of Rheumatology/European League against Rheumatism collaborative initiative. *Arthritis & Rheumatism*.

[5] Ayzenberg O., Reiff D. B., Levin L. (1983). Bilateral pneumothoraces and pleural effusions complicating rheumatoid lung disease. *Thorax*.

[6] Rubin E. H., Gordon M., Thelmo W. L. (1967). Nodular pleuropulmonary rheumatoid disease: report of two cases and review of literature. *American Journal of Medicine*.

[7] Crisp A. J., Armstrong R. D., Grahame R., Dussek J. E. (1982). Rheumatoid lung disease, pneumothorax and eosinophilia. *Annals of the Rheumatic Diseases*.

[8] Tahir H., Allard S. A., Jawed S. (2001). Spontaneous hydropneumothorax in a man with rheumatoid arthritis. *Rheumatology*.

[9] Rguibi M., Abouzaher M., Kherass B. (2003). Rheumatoid lung nodules with hydropneumothorax. *Joint Bone Spine*.

[10] Steeghs N., Huizinga T. W. J., Dik H. (2005). Bilateral hydropneumothoraces in a patient with pulmonary rheumatoid nodules during treatment with methotrexate. *Annals of the Rheumatic Diseases*.

[11] Ziadé M., Van Linthoudt D., Ris H. B., So A. K. L. (2008). Toux sèche, douleurs thoracíques et dyspnée chez un patient atteint d’une polyarthrite rhumatoïde. *Praxis*.

[12] Sassoon C. S., Light R. W. (1985). Chylothorax and pseudochylothorax. *Clinics in Chest Medicine*.

[13] Lama A., Ferreiro L., Toubes M. E. (2016). Characteristics of patients with pseudochylothorax—a systematic review. *Journal of Thoracic Disease*.

[14] Garcia-Zamalloa A., Ruiz-Irastorza G., Aguayo F. J., Gurrutxaga N. (1999). Pseudochylothorax: report of 2 cases and review of the literature. *Medicine*.

[15] Wrightson J. M., Stanton A. E., Maskell N. A., Davies R. J., Lee Y. C. (2009). Pseudochylothorax without pleural thickening: time to reconsider pathogenesis?. *Chest*.

[16] Muresan C., Muresan L., Grigorescu I., Dumitrascu D. L. (2015). Chyliform effusion without pleural thickening in a patient with rheumatoid arthritis: a case report. *Lung India*.

[17] Molina L. Z., Redondo G. M., Borbolla A. M. (2016). Seudoquilotórax sin engrosamiento pleural associado a artritis reumatoide. *Archivos de Bronconeumología*.

